# Echocardiographic Assessment of Ventricular Function in Young Patients with Asthma

**DOI:** 10.5935/abc.20180052

**Published:** 2018-03

**Authors:** Camilla Rayane De-Paula, Giselle Santos Magalhães, Nulma Souto Jentzsch, Camila Figueredo Botelho, Cleonice de Carvalho Coelho Mota, Tatiane Moisés Murça, Lidiana Fatima Correa Ramalho, Timothy C. Tan, Carolina Andrade Braganca Capuruço, Maria da Gloria Rodrigues-Machado

**Affiliations:** 1 Faculdade Ciências Médicas - Minas Gerais (FCM-MG), Belo Horizonte, MG - Brazil; 2 Universidade Federal de Minas Gerais (UFMG), Belo Horizonte, MG - Brazil; 3 Universidade Salgado de Oliveira, Belo Horizonte, MG - Brazil; 4 Prefeitura de Belo Horizonte - Unidade de Referência Secundária Saudade, Belo Horizonte, MG - Brazil; 5 Clínica Conrad, Belo Horizonte, MG - Brazil; 6 Westmead Hospital - Faculty of Medicine - University of Sydney, Sidney - Austrália; 7 Blacktown Hospital - Faculty of Medicine - University of Western Sydney, Sidney - Austrália; 8 School of Medical Sciences - Faculty of Medicine - University of New South Wales, Sidney - Austrália

**Keywords:** Exertional Dyspnea / physiopathology, Echocardiography, Doppler, Asthma / physiopathology, Vascular Remodeling, Ventricular Dysfunction

## Abstract

**Background:**

Despite significant advances in understanding the pathophysiology and management of asthma, some of systemic effects of asthma are still not well defined.

**Objectives:**

To compare heart function, baseline physical activity level, and functional exercise capacity in young patients with mild-to-moderate asthma and healthy controls.

**Methods:**

Eighteen healthy (12.67 ± 0.39 years) and 20 asthmatics (12.0 ± 0.38 years) patients were enrolled in the study. Echocardiography parameters were evaluated using conventional and tissue Doppler imaging (TDI).

**Results:**

Although pulmonary acceleration time (PAT) and pulmonary artery systolic pressure (PASP) were within normal limits, these parameters differed significantly between the control and asthmatic groups. PAT was lower (p < 0.0001) and PASP (p < 0.0002) was higher in the asthma group (114.3 ± 3.70 ms and 25.40 ± 0.54 mmHg) than the control group (135.30 ± 2.28 ms and 22.22 ± 0.40 mmHg). The asthmatic group had significantly lower early diastolic myocardial velocity (E', p = 0.0047) and lower E' to late (E'/A', p = 0.0017) (13.75 ± 0.53 cm/s and 1.70 ± 0.09, respectively) compared with control group (15.71 ± 0.34 cm/s and 2.12 ± 0.08, respectively) at tricuspid valve. In the lateral mitral valve tissue Doppler, the asthmatic group had lower E' compared with control group (p = 0.0466; 13.27 ± 0.43 cm/s and 14.32 ± 0.25 cm/s, respectively), but there was no statistic difference in the E'/A' ratio (p = 0.1161). Right isovolumetric relaxation time was higher (p = 0.0007) in asthmatic (57.15 ± 0.97 ms) than the control group (52.28 ± 0.87 ms), reflecting global myocardial dysfunction. The right and left myocardial performance indexes were significantly higher in the asthmatic (0.43 ± 0.01 and 0.37 ± 0.01, respectively) compared with control group (0.40 ± 0.01 and 0.34 ± 0.01, respectively) (p = 0.0383 and p = 0.0059, respectively). Physical activity level, and distance travelled on the six-minute walk test were similar in both groups.

**Conclusion:**

Changes in echocardiographic parameters, evaluated by conventional and TDI, were observed in mild-to-moderate asthma patients even with normal functional exercise capacity and baseline physical activity level. Our results suggest that the echocardiogram may be useful for the early detection and evoluation of asthma-induced cardiac changes.

## Introduction

Asthma is characterized by chronic inflammation and remodeling of the airways.^1^ This remodeling leads to structural changes in the walls of the airways induced by repeated injury and repair, which can cause an irreversible loss of lung function.^2^ Moreover, asthma can lead to an increase in bronchial angiogenesis^3^ and remodeling of the pulmonary vessels, culminating in changes in both bronchial and pulmonary circulation.^4^

The interaction between respiratory diseases and cardiovascular function is complex. Changes in the structure and function of the right ventricle are associated with pulmonary hypertension.^5^ Recurring hypoxemia and hypercapnia associated with different mediators and cytokines related to chronic inflammation of the airways in patients with asthma cause pulmonary vasoconstriction and the development of pulmonary hypertension, with the consequent hypertrophy/dilatation of the right ventricle.^6^ Diastolic dysfunction of the right ventricle is the earliest hemodynamic change found in patients with asthma due to the increase in the afterload imposed on the ventricle.^7^ Pulmonary disease affects the size, shape and function of the right ventricle, but altered respiratory function can also affect the left ventricle.^5^

Echocardiography is a non-invasive, relatively safe, cost effective and easily accessible method for the right ventricle assessment. Tissue Doppler imaging (TDI) has been demonstrated to furnish a quantitative measure of regional velocities in the myocardium as well as systolic and diastolic intervals.^8^ TDI can detect subclinical abnormalities of the right ventricle in a phase when conventional echocardiographic findings are still within normal ranges,^8^ thereby enabling the detection of right ventricular dysfunction in the early stage of a disease.^9-11^

Recent studies on young adults with asthma have employed TDI and found subclinical diastolic dysfunction directly related to the severity of the condition, suggesting that this imaging technique has greater predictive value than conventional echocardiography for the evaluation of right ventricular function^9^. Similar findings have been reported for children and adolescents in the stable phase of asthma. Shedeed et al.^11^ evaluated children and adolescents aged 5 to 15 years with mild to severe asthma and found that TDI demonstrated right ventricular dysfunction that was positively correlated with the severity of the condition, despite the conventional echocardiogram being apparently normal. Likewise, Ozdenir et al.^10^ found a negative correlation between right ventricular dysfunction and peak expiratory flow in children with asthma, suggesting that TDI has important diagnostic value for the early detection and monitoring of heart repercussions in children with asthma.

The clinical phenotype of asthma can differentially affect myocardial performance. Children with asthma and a predominance of shallow breathing exhibit more severe myocardial dysfunction than those with a predominance of wheezing as the manifestation of the condition.^12^ The aim of the present study was to compare heart function, quality of life, physical activity level, functional exercise capacity and inspiratory muscle strength/endurance in young patients with mild-to-moderate asthma and healthy controls to determine the impact of this condition on echocardiographic variables.

## Methods

### Study population

Male and female children and adolescents from 10 to 16 years with mild to moderate asthma were enrolled in the present study. Asthma severity was established based on the guidelines of the Global Strategy for Asthma Management and Prevention.^13^ The control group comprised of children and adolescents considered healthy. The groups were matched for sex and age.

### Inclusion criteria

The group with asthma comprised children and adolescents with clinical and spirometric diagnosis of asthma, for more than 30 days with no history of acute upper or lower airway infection or exacerbation of the condition. The participants in the control group had a normal clinical history and normal lung function.

### Exclusion criteria

Children and adolescents with acute or chronic lung disease, major thoracic deformities, neuromuscular, cardiovascular, digestive, rheumatic, osteoarticular or genital-urinary disorders, genetic syndromes or any adverse health conditions that limited the safe performance of the tests proposed in the protocol were excluded from the study.

### Evaluation protocol

Height (meters) and weight (kilograms) were measured using an anthropometric scale (Filizola^TM^, São Paulo, SP, Brazil). The tests were conducted in two steps. Step 1 - spirometric analysis was performed to confirm the diagnosis and classification of asthma. Next, the quality of life and baseline physical activity questionnaires were administered. The strength and endurance of the inspiratory muscles were then measured. Functional capacity was evaluated using the six-minute walk test (6MWT) 30 minutes after the evaluation of the inspiratory muscles. Step 2 - Echocardiography was performed. The order of the two steps was determined randomly and a maximum interval of 15 days was respected between steps.

### Pulmonary function test

The spirometric variables analyzed were forced vital capacity (FVC), forced expiratory volume in the first second of FVC (FEV_1_) and the Tiffeneau index (FEV_1/_FVC). The group with asthma was also submitted to the bronchodilator test 20 minutes after the inhalation of salbutamol (400 µg/dose) distributed in four inhalations of 100 µg with a one-minute period between inhalations. Predicted values were analyzed and described using the equations proposed by Polgar and Promadhat.^14^ An increase in FEV_1_ equal to or greater than 12% of the predicted after the administration of salbutamol confirmed the variable limitation to airflow.

### Evaluation of maximum inspiratory pressure

Maximum inspiratory pressure (MIP) was evaluated using an analog pressure gauge (MDI® model MVD300, Porto Alegre, Brazil) beginning from residual volume. At least five reproducible measurements were taken and the maneuvers were repeated until the two highest measurements did not differ by more than 5%.^15^ The highest measurement was used to establish the load for the evaluation on inspiratory muscle endurance.

### Evaluation of inspiratory muscle endurance

Inspiratory muscle endurance was evaluated after the determination of MIP, using a modified version of the protocol proposed by Sette et al.^16^ Inspiratory muscle endurance was defined as the maximum time tolerated of spontaneous breathing with a load corresponding to 30% of MIP until exhaustion, which was defined as the inability to overcome inspiratory resistance in two consecutive attempts. The criteria for interrupting the test were intense weariness, dizziness, discomfort, cheek pain or peripheral oxygen saturation (SpO_2_) less than 85%.

### Evaluation of functional capacity using the 6MWT

The 6MWT was performed based on the guidelines of the American Thoracic Society. The participant was instructed to walk as fast as possible along a 30-meter flat corridor marked every three meters and received standardized verbal encouragement every 30 seconds by the same evaluator. A second evaluator remained at one of the extremities of the track to assist in the data collection at the beginning and end of the test. Blood pressure (BP), respiratory rate (RR), heart rate (HR), SpO_2_, and the Borg dyspnea score at rest and during exertion were the variables measured at the beginning and end of the test. The 6MWT was performed twice, with a 30-minute rest interval between tests. The test on which the participant travelled the longer distance was considered in the statistical analysis. The criteria for interrupting the test were extreme weariness, SpO_2_ less than 85% or any other discomfort. The participant was told that the test could be interrupted at any type if he/she felt any discomfort. Dyspnea at rest and during exertion was evaluated using the modified Borg scale,^17^ which is scored from 0 to 10 points based on verbal responses that correspond to no or maximum shortness of breath, respectively.

### Evaluation of quality of life

Quality of life was evaluated using the Pediatric Quality of Life Inventory^TM^ version 4.0 (PedsQL 4.0).^18,19^ Self-assessments were available for the following age groups: 5 to 7, 8 to 12 and 13 to 18 years. The items on the forms for each age group are similar, differing only in terms of the use of language adequate to the level of development. The quality of life of the group with asthma was also evaluated using the Paediatric Asthma Quality of Life Questionnaire (PAQLQ),^20^ which has been translated and culturally adapted to Brazilian Portuguese for children and adolescents aged 7 to 17 years.^21^

### Evaluation of baseline physical activity

Physical activity was evaluated using the Physical Activity Questionnaire - Child (PAQ-C),^22^ which measures the level of physical activity of children and adolescents in the previous week.

### Evaluation of echocardiographic variable

A single pediatric cardiologist who was blinded to the respiratory status of the participants performed the echocardiogram. The exam was performed with the participant positioned in left lateral and dorsal decubitus. The Toshiba echocardiograph was used with variable frequency transducers from 2.0 to 7.0 MHz. At least five consecutive beats were obtained from the parasternal window to determine the inner diameters of the ventricles. The exams were recorded and analyzed offline by two specialists in pediatric echocardiography.

The left and right ventricular functions were assessed by two-dimensional echocardiography: M-mode, color-flow imaging, standard pulsed-wave Doppler and TDI, according to guidelines of the American Society of Echocardiography. The following data were collected for statistical evaluation: measurements of aortic dimension, left atrium, right ventricle anterior wall, right ventricular end-diastolic dimension, interventricular septum, left ventricular end-diastolic dimension, left ventricular systolic dimension and left ventricle posterior wall obtained by the M mode paraesternal long and short axis view. There was no patient with congenital heart disease and all of them had symmetric left ventricular systolic function.

**Figure 1:**
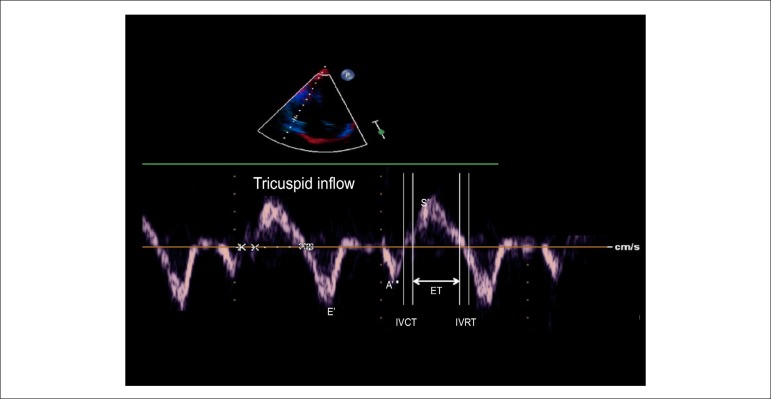
Tissue Doppler imaging performed at tricuspid annulus in apical 4-chamber view (E'- peak early diastolic annular tricuspid velocity; A': peak late diastolic annular tricuspid velocity; S': systolic annular velocity; IVRT: isovolumetric relaxation time; IVCT: isovolumetric contraction time; ET: ejection time)

The apical four-chamber view enables studying blood inflow through the atrioventricular valves. The early (E) and late (A) diastolic velocities of mitral and tricuspid valves and E/A ratio were used to evaluate biventricular filling function. TDI was used to evaluate cardiac load and determine the myocardial performance index (MPI). The left ventricular TDI was achieved at the lateral wall through the mitral annulus, whereas the right ventricular TDI was achieved through the tricuspid lateral annulus. The recordings of peak early (E') and late (A') diastolic velocities, E´/A´ ratio, systolic (S´) annular velocity, isovolumetric relaxation time (IVRT) and isovolumetric contraction time (IVCT) were obtained in the apical four-chamber view. MPI is defined as the IVCT and IVRT divided by the ejection time (ET) (Figure 1).

The right ventricular systolic function was assessed by fractional area change (FAC), derived from tricuspid lateral annular systolic velocity wave (S') and tricuspid annular plane systolic excursion (TAPSE).

**Figure 2:**
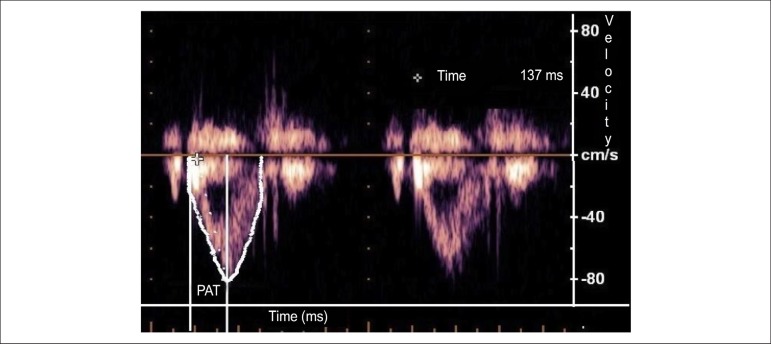
Pulsed-wave Doppler of pulmonary artery (PAT: pulmonary acceleration time = interval from onset of pulmonary flow to peak velocity; shorter acceleration time = higher pulmonary arterial pressure)

Pulmonary systolic arterial pressure (PSAP) was also estimated using two methods. Pulsed-wave Doppler tracing across the pulmonary valve was performed using the pulmonary acceleration time (PAT) by left parasternal short-axis view (Figure 2). The normal profile is symmetrical in shape. When pulmonary pressure and pulmonary vascular resistance are high, the peak occurs earlier. The other method was measuring maximal tricuspid regurgitation velocity, applying the modified Bernoulli equation to convert this value into pressure values and adding the estimated right atrial pressure (RAP). Normal RAP was considered 5 mmHg. PSAP = tricuspid regurgitation gradient + RAP. PSAP = (Vmax² x 4) + RAP. Normal systolic arterial pressure is up to 30 mmHg at rest and up to 40 mmHg during exercise.

### Sample size

In order to calculate the sample size, we considered as objective to test the equality of the means of the mitral E-wave velocity among the groups of asthmatic patients and controls.^23^ In order to detect a minimum difference of 4.7 cm/s between means, with a significance of 5%, minimum power of 80%, and variance based on a previous study,^9^ it was necessary to obtain a ratio of 0.9 between controls/asthmatics, corresponding to 20 asthmatic children and 18 controls.

### Statistical analysis

The Kolmogorov-Smirnov test was used to determine the normality of the data. The variables were expressed as central tendency (mean and median) and variability (standard error of the mean or interquartile range-IQR). When appropriate, either the non-paired t-test or the Mann-Whitney test was used for the comparison of the different variables analyzed, and either Pearson's or Spearman's correlation coefficient were calculated to evaluate associations between the independent variables and response variable. All analyses were performed with the GraphPad Prism software (version 5.0, GraphPad Software, Inc., La Jolla, CA, USA). A p-value < 0.05 was considered indicative of statistical significance.

## Results

### Anthropometric and pulmonary function test data

Control and asthmatic groups were similar with regard to age, weight, height, and body mass index (BMI). FEV_1_ and the Tiffeneau index (FEV_1_/FVC) were significantly lower in the group with asthma than the control group (Table 1). All asthmatic patients were clinically stable. Of the 20 patients, 86.95% presented mild asthma, 8.70% moderate asthma and 4.35% very severe asthma.

**Table 1:** Anthropometric data, and pulmonary function test in control and asthmatic groups

Variable	Control (n = 18)	Asthma (n = 20)	p
Age (years)	12.67 ± 0,39	12.0 ± 0.38	0.143^M^
Male sex	44. 44%	50%	-
Weight (Kg)	52.5 ± 5.0	50.3 ± 3.2	0.21^T^
Height (m)	1.57 ± 0.34	1.53 ± 0.22	0.44^T^
BMI	21.1 ± 1.4	21.0 ± 1.0	0.93^T^
Normal weight range	44.45%	47.6%	-
Overweight	33.33%	23.8%	-
Obese	22.22%	28.6%	-
**Pulmonary function**			
FVC (% predicted)	108.7% ± 4.7	95.8% ± 3.1	0.814^T^
FEV_1_ (% predicted)	102.2% ± 4.9	84.4% ± 3.5	0.011^T^
FEV_1 _/FVC (%)	95.7% ± 1.6	86.4% ± 2.9	0.027^T^

Data expressed as mean ± SEM. BMI: body mass index; FVC: forced vital capacity; FEV_1_: forced expiratory volume in first second of FVC; FEV_1_/FVC: Tiffeneau index. Comparison between the two groups was made through the t-student or non-parametric statistical test Mann-Whitney test.

T t-Student and

M Mann-Whitney.

### Echocardiographic characteristics

In the present study, conventional and tissue echocardiographic parameters in healthy and asthmatic children and adolescents free of any cardiovascular symptoms were assessed.

TAPSE, FAC% and S' were similar in both asthmatic and control groups. MPI was higher in asthmatic group. TDI of right diastolic function revealed that E', A' and the E'/A' ratio, evaluated in the tricuspid annulus, differed significantly between groups. Similarly, E and A diastolic annular tricuspid velocity and E/A differed significantly between groups (Table 2). Moreover, the IVRT was significantly (p = 0.0007) higher in asthmatic group (57.15 ± 0.97 ms) in relation to control (52.28 ± 0.87 ms) group.

**Table 2:** Doppler echocardiogram parameters of right ventricle systolic and diastolic function in control and asthmatic groups

Variable (normal value)	Systolic function		p
	Control (n = 18)	Asthma (n = 20)	
TAPSE cm ( > 1.6)	1.9 ± 0.19	1.8 ± 0.11	0.184^M^
FAC % ( > 35)	40 ± 3.21	38 ± 2.89	0.212^M^
S' cm/s ( > 9.5)	12.29 ± 0.26	11.67 ± 0.34	0.3342^M^
MPI ( < 0.55)	0.40 ± 0.01	0.43 ± 0.01	0.0383^M^
**Diastolic function**			
Tricuspid E'/A' ratio ( > 0.52)	2.12 ± 0.08	1.70 ± 0.09	0.0017^T^
Tricuspid E' cm/s ( > 7.8)	15.71 ± 0.34	13.75 ± 0.53	0.0047^T^
Tricuspid E/A ratio ( > 0.8)	2.34 ± 0.09	1.71 ± 0.06	< 0.0001^T^

Data expressed as mean ± SEM. TAPSE: tricuspid annular plane systolic excursion; FAC %: fractional area change; S': systolic myocardial velocity; MPI: myocardial performance index; E': early diastolic myocardial velocity; A': late diastolic myocardial velocity; E: peak early diastolic annular tricuspid velocity; A: peak late diastolic annular tricuspid velocity (atrial contraction). Data analysis for comparison between the two groups was made through the t-student or non-parametric statistical test Mann-Whitney test.

T t-Student and

M Mann-Whitney. In the tricuspid valve, the value of MPI is 0.39 (0.6) and 0.43 (0.6) for control and asthmatic groups.

Although PAT and PASP were within normal limits (> 130 ms and < 35 mmHg, respectively), these parameters differed significantly between the control and asthmatic groups. PAT was lower (p < 0.0001) and PASP (p < 0.0002) was higher in the asthma group (114.3 ± 3.70 ms and 25.40 ± 0.54 mmHg) than the control group (135.30 ± 2.28 ms and 22.22 ± 0.40 mmHg).

Table 3 shows that S' was lower and the MPI was higher in the group with asthma. TDI of left diastolic function revealed that both E' and A' differed significantly between groups. Peak E and A diastolic annular mitral velocity and E/A differed significantly between groups.

**Table 3:** Doppler echocardiogram parameters of left ventricle systolic and diastolic function in control and asthmatic groups

Variable (normal value)	Systolic function		p
	Control (n = 18)	Asthma (n = 20)	
Ejection fraction % (> 35)	69.0 ± 0.47	69.0 ± 0.80	0.4677^T^
Lateral mitral S' cm/s (> 6.7)	8.01 ± 0.20	7.30 ± 0.21	0.0170^M^
MPI (< 0.55)	0.34 ± 0.01	0.37 ± 0.01	0.0059^T^
**Diastolic function**			
Lateral mitral E'/A' ratio (> 0.82)	2.89 ± 0.09	2.52 ± 0.20	0.1161^T^
Lateral mitral E' cm/s (> 10.0)	14.32 ± 0.25	13.27 ± 0.43	0.0466^T^
Mitral E/A ratio (> 0.8)	3.42 ± 0.17	2.25 ± 0.14	< 0.0001^T^

Data expressed as mean ± SEM. S': systolic myocardial velocity; MPI: myocardial performance index; E': early diastolic myocardial velocity; A': late diastolic myocardial velocity; E: peak early diastolic annular mitral velocity; A: peak late diastolic annular mitral velocity (atrial contraction). Data analysis for comparison between the two groups was made through the t-student or non-parametric statistical test Mann-Whitney test.

T t-Student and

M Mann-Whitney. In the mitral valve, the value S' is 7.93 (1.07) and 7.12 (1.08) for control and asthmatic groups.

Among the 18 healthy children and adolescents submitted to echocardiograms, nine were submitted to an evaluation of inspiratory muscle endurance and functional capacity, along with the administration of the questionnaires on quality of life and level of physical activity. The group with asthma was submitted to all tests employed in the present study.

### Inspiratory muscle endurance

No significant differences between control and asthma groups were found regarding MIP (109.4 ± 14.19 cmH_2_O and 92.14 ± 5.62 cmH_2_O, p = 0.178) or baseline dyspnea (0.14 ± 0.09 and 0.18 ± 0.10, p = 0.871). Despite the shorter respiratory muscle endurance time in the group with asthma (128.9 ± 14.08 s), the difference did not achieve statistical significance in comparison with control group (154.9 ± 46.69 s). Final Borg scale scores were significantly higher in comparison to the baseline evaluation in both groups. Moreover, the group with asthma (6.1 ± 0.39) had significantly (p = 0.0129) higher final Borg scores in comparison to the control group (3.67 ± 0.41).

### Baseline physical activity and functional exercise capacity

Mean baseline physical activity, evaluated by PAQ-C, was similar (p = 0.65) in both control (2.2) and asthmatic (2.04) groups. The mean number of hours spent in front of the television per day was five hours in the control group and 5.71 hours in the group with asthma.

The functional exercise capacity was evaluated by 6MWT. All participants completed the test without interruption. No significant differences between groups were found regarding the cardiopulmonary variables (BP, HR, SpO_2_, and dyspnea). The walked distance did not differ (p = 0,239) between control (327.3 ± 15.73 m) and asthma (328.8 ± 8.61 m) groups.

### Evaluation of quality of life

The quality of life in control and asthmatic groups was measured using the PedsQL 4.0. No significant difference between groups was found regarding the mean total PedsQL 4.0 score (p = 0.418) or separately the scores on the emotional (p = 0.698), social (p = 0.730), school functioning (p = 0.626) and psychosocial (p = 0.984) domains. The score on the physical domain, however, was significantly lower (p = 0.005) in the group with asthma (74.06 ± 2.54) in comparison to the control group (92.86 ± 3.71). Regarding the PAQLQ, no significant differences between sexes were found on any of the domains. The "symptoms" domain had the greatest negative impact (5.22 ± 0.23).

## Discussion

The present findings demonstrate for the first time that PAT was significantly lower and PSAP was significantly higher in the group with asthma compared to the controls. TDI has been used to evaluate quantitative measurements of regional velocities of the myocardium and both the systolic and diastolic intervals.^8^ TDI enables the detection of right ventricular dysfunction in the early stages of respiratory disease.^9^ In the current study, significant differences between groups were found regarding E' and A' evaluated in the tricuspid and mitral annuli. In addition, the MPI of the right and left ventricles was significantly higher in the group with asthma. Interestingly, respiratory muscle performance, baseline physical activity level and exercise capacity were similar in both groups. Taken together, these findings suggest that echocardiographic parameters, especially TDI parameters, can be useful as a complementary evaluation for patients with asthma, allowing the early detection of repercussions on the heart.

The interaction between respiratory diseases and cardiovascular function is complex. Changes in the structure and function of the right ventricle are associated with pulmonary hypertension.^5^ In the present study, although the conventional echocardiogram demonstrated no evidence of changes in the structure of the right ventricle, the group with asthma exhibited a reduction in PAT and an increase in PSAP in relation to control group. Recent study has demonstrated that PAT inversely correlates with right heart catheterization-measured pulmonary hemodynamics and directly correlates with pulmonary arterial compliance in children.^24^ Unlike findings described in studies by Shedeed et al.,^11^ Ozdemir et al.^10^ and Zedan et al.,^12^ no right ventricular hypertrophy was found in the group with asthma in the present investigation. Moreover, in the current study the conventional Doppler echocardiogram revealed statistically significant difference between the controls and the group with asthma regarding peak velocities during the early diastole and atrial contraction (E, A and E/A) evaluated in the annulus of the mitral and tricuspid valves. In contrast, Shedeed et al.^11^ found no significant differences in these variables between controls and a group with asthma or between the different degrees of asthma severity.

A number of studies have demonstrated that patients with asthma exhibit diastolic dysfunction.^9,11,12^ Indeed, in the current study significant differences between the controls and the group with asthma were found regarding myocardial diastolic velocities E' and A' as well as the E'/A' ratio evaluated in the tricuspid annulus. Similar results were found in the mitral valve annulus, with a reduction in myocardial velocity during early diastole and an increase in myocardial velocity during atrial contraction. A significant increase in the IVRT was also found in the group with asthma, contributing to a significant increase in the MPI. In contrast, the increase in the MPI of the left ventricle occurred at the cost of a reduction in systolic velocity in the ventricle.

The clinical phenotype of asthma may differentially affect myocardial performance. Zedan et al.^12^ compared the MPI of children with asthma according to the phenotype (predominance of shallow breathing or wheezing as the clinical manifestation) and found that those with shallow breathing had a higher MPI. In the present study, the asthmatic children and adolescents were evaluated in a single group based only on the clinical and spirometric diagnosis of asthma.

In the current study, MIP was similar in both groups, despite the significant reductions in FEV_1_ and Tiffeneau index in the asthmatic group. The results of studies involving inspiratory muscle strength in asthmatic children and adolescents are conflicting. Some studies show that there is no difference,^25,26^ and other studies show that the strength of the inspiratory muscles of children and adolescents with asthma is reduced relative to their peers.^27^ Similar results were observed in inspiratory muscle endurance. Endurance test was similar in the control and asthmatic groups. However, exertion dyspnea evaluated at the end of the endurance test was significantly more intense in the group with asthma, suggesting that this variable may have a discriminative value between healthy individuals and those with asthma when submitted to the same level of inspiratory muscle overload. There is a number of determinant factors of inspiratory muscle endurance, such as contraction strength and duration, shortening velocity, the relationship between baseline inspiratory pressure (IP) and MIP (IP/MIP) and the inspiratory flow pattern adopted by patients during the evaluation.^28^ Further studies are needed to clarify the greater shortness of breath in the group with asthma.

The 6MWT is considered a safe, easy-to-administer method for the evaluation of sub-maximum exercise capacity in healthy children and adolescents,^29^ as well as those with respiratory diseases.^30-32^ Similar to the results of Basso et al.^31^ and Soares et al.,^27^ in the present study, no significant difference between groups was found regarding the distance walked or the cardiovascular variables analyzed before and after the 6MWT. In the same way, studies using other functional capacity assessment methods, such as the shuttle walking test^33^ and cardiopulmonary exercise test^34^ also did not observe a difference between asthmatic children and adolescents and control group.

Quality of life is one of the most important outcomes in the evaluation of patients with chronic disease. In the present study, this aspect was evaluated using a generic questionnaire as well as a specific questionnaire for children and adolescents with asthma. Regarding the generic questionnaire, quality of life was similar in both groups with regard to most domains, except the score on the physical domain, which was significantly lower in the group with asthma. In agreement with data described by Basaran et al.^35^ and Andrade et al.,^30^ the mean score on the asthma-specific questionnaire was 5.67 ± 0.23, which indicates a good quality of life among the children and adolescents studied in the present investigation.

### Limitations of the study

The small sample size could be considered a limitation of the present study. However, even with the small number of participants, it was possible to demonstrate changes in conventional and tissue echocardiographic variables among the children and adolescents with asthma in comparison to the control group. Another limitation regards the evaluation of functional capacity. The 6MWT is considered a sub-maximal exercise test for measuring physical functional capacity. It is possible that the variables analyzed on a maximum cardiopulmonary stress test would be more sensitive in detecting differences in functional capacity between individuals considered healthy and those with asthma. A third limitation was the failure to evaluate the breathing pattern adopted during the respiratory muscle endurance test. The record of inspiratory flow allows the evaluation of inspiratory time, expiratory time, total cycle and the inspiratory time/total cycle ratio, as performance on the endurance test can vary depending on the breathing pattern adopted. The precise mechanism to clarify the difference in exertion dyspnea between healthy individuals and those with asthma during the inspiratory muscle endurance test needs to be determined.

## Conclusion

Patients with asthma presented significant changes in diastolic velocities of the myocardium and the MPI of both ventricles, but with no repercussions regarding exercise capacity evaluated using the 6MWT. Further studies are needed to confirm these findings and to evaluate the clinical implications of these abnormalities.
